# Binge eating behaviours are associated with recurrent weight gain after metabolic and bariatric surgery

**DOI:** 10.1111/cob.12735

**Published:** 2025-01-27

**Authors:** Matthew Cali, Deepali K. Ernest, Luyu Xie, Jeffrey N. Schellinger, M. Sunil Mathew, Aparajita Chandrasekhar, Jane Guo, Gloria L. Vega, Sarah E. Messiah, Jaime P. Almandoz

**Affiliations:** ^1^ University of Texas Southwestern Medical School Dallas Texas USA; ^2^ University of Texas Health Science Center, School of Public Health Houston Texas USA; ^3^ Center for Pediatric Population Health, UTHealth School of Public Health Dallas Texas USA; ^4^ University of Texas Health Science Center, School of Public Health Dallas Texas USA; ^5^ Department of Internal Medicine, Division of Endocrinology University of Texas Southwestern Medical Center Dallas Texas USA; ^6^ Department of Clinical Nutrition University of Texas Southwestern Medical Center Dallas Texas USA

**Keywords:** binge eating behaviours, food addiction, metabolic bariatric surgery, recurrent weight gain

## Abstract

**Background:**

Recurrent weight gain (RWG) is a major post‐operative challenge among metabolic and bariatric surgery (MBS) patients. Binge eating behaviours (BEB) and food addiction (FA) have been identified as significant predictors of post‐MBS RWG. However, limited research has investigated their independent associations with post‐MBS RWG.

**Methods:**

This cross‐sectional study collected data via a self‐reported questionnaire of post‐MBS patient demographics and eating behaviours from a single‐site academic obesity medicine program. The Binge Eating Scale and Yale Food Addiction Scale 2.0 collected data on BEB and FA exposure variables, respectively. ANOVA/chi‐square tests determined bivariate associations with BEB and FA, while multivariable logistic regression models examined independent adjusted associations of BEB and FA with RWG% cut‐offs.

**Results:**

Of the 294 MBS patients (90.48% female, and 51.71% non‐Hispanic white), 42.3% had BEB, 12.55% had severe FA, 7.36% moderate FA, and 7.36% mild FA. After adjustment, BEB was significantly associated with all magnitudes of post‐MBS RWG, with the highest odds observed at 50% RWG [OR = 3.07; 95% CI: 1.45, 6.49; *p* = 0.003]. FA was not significantly associated with post‐MBS RWG.

**Conclusion:**

Results showed that BEB, but not FA, was associated with post‐MBS RWG. MBS patient support teams should consider screening for BEB at post‐MBS visits.

**What is known about this subject**

- Metabolic and bariatric surgery (MBS) is an effective treatment for obesity and many of the health benefits correlate with the magnitude of weight reduction.
- Binge eating behaviours and food addiction are associated with recurrent weight gain post‐MBS.

**What this study adds**

- Results show that there is a high prevalence of binge eating behaviours after bariatric surgery.
- Post‐MBS patients with higher scores on the Binge Eating Scale had higher odds of recurrent weight gain, while those with higher scores on the Yale Food Addiction Scale did not.
- Clinicians should consider screening post‐MBS patients for health behaviours that are associated with adverse weight and health outcomes.

## 1 INTRODUCTION

Obesity is a worldwide health crisis that currently impacts over 3 billion people.^1^ In the last few decades, the global prevalence of obesity has nearly tripled,^2^ which is cause for concern given that obesity is a risk factor for many chronic diseases like type 2 diabetes, heart disease, and certain cancers.^2^ Obesity is also associated with decreased quality of life and a shortened lifespan among affected individuals.^3^, ^4^ Specifically in the United States (US), the prevalence of obesity has risen from 30.5% to 41.9% between 2017 and 2020, translating to an economic and social impact burden of more than 1.4 trillion US dollars per year.^5^, ^6^, ^7^

There are many strategies for treating the chronic and complex health condition of obesity. These strategies include positive lifestyle changes, anti‐obesity medications (AOM), and metabolic and bariatric surgery (MBS). MBS is one of the most effective and safe treatments for obesity, and it is estimated that over 480 000 of these surgeries are performed annually around the world.^8^ Despite the above outlined weight management strategies, recurrent weight gain (RWG) remains a common‐post MBS concern.^9^ The resolution of many obesity‐related complications is associated with the magnitude of weight loss following MBS.^10^ RWG is a cause for concern as it can contribute to the exacerbation or recurrence of complications, including type 2 diabetes (T2D),^11^ mental health disorders such as depression,^12^ and decreased quality of life.^13^

Maladaptive eating patterns and disordered eating, specifically binge eating and food addiction (FA), can significantly contribute to post‐bariatric RWG.^14^ FA is defined as an uncontrolled consumption of foods high in fats or carbohydrates, which is triggered by the sight, sound, or smell of palatable foods.^15^, ^16^, ^17^ While there is currently no Diagnostic and Statistical Manual of Mental Disorders (DSM) criteria for FA, healthcare professionals have advocated for FA to be classified similarly to other substance use disorders (SUD) due to the addiction‐like presentation of FA.

Binge eating disorder (BED) is characterized by the consumption of a large quantity of food in a short span of time with a sense of loss of control when eating.^18^ According to the 5th edition of the DSM (DSM‐5), a binge eating episode must occur on average at least 1 day a week for 3 months to meet the diagnostic criteria for BED.^18^ A history of BEB is common among MBS‐seeking patients, with approximately 15.7% to 26.6% reporting behaviours consistent with BEB,^19^ which may be associated with unfavourable surgical outcomes.^20^

Although several studies have highlighted an overlap in BEB and FA,^21^ recent studies have underscored several important diagnostic and behavioural distinctions of each condition.^21^, ^22^ A more profound understanding of these differences between BEB and FA in a post‐MBS population can better inform healthcare providers in identifying groups of individuals at risk for post‐MBS RWG and inform the tailoring of existing treatments to support individuals at risk. Therefore, this study aimed to assess the prevalence of BEB and FA in a post‐MBS sample in the US and examine the degree to which each condition is associated with post‐MBS RWG.

## 2 METHODS

This cross‐sectional study analysed self‐reported survey data from patients enrolled in the Weight Wellness obesity medicine clinic at UT Southwestern Medical Center in Dallas, Texas. Participants who had never completed an MBS procedure or had missing information on individual BEB and FA behaviours were excluded from the analysis. The final analytical sample consisted of 294 MBS participants.

### 2.1 Main exposure variables

We evaluated BEB and FA as independent exposures.

#### 2.1.1 Binge eating behaviours

BEB was assessed using the 16‐item Binge Eating Scale (BES) developed by Gormally et al.^23^ The scale ranged between 0 and 46, with a BES score of <15 indicating no BEB and a score of ≥15 indicating the presence of BEB.^24^, ^25^

#### 2.1.2 Food addiction

FA was assessed using the 13‐item Yale Food Addiction Scale 2.0 (YFAS 2.0).^22^ Using the appropriate scoring system detailed elsewhere,^26^ a composite categorical FA exposure variable was created, where 1 = no FA, 2 or 3 = mild FA, 4 or 5 = moderate FA, and ≥6 = severe FA.

### 2.2 Main outcome variable

The main outcome variable, percent recurrent weight gain (RWG%), was calculated using the following formula:

$$\left[\left(\text{current weight}-\text{postop nadir weight}\right)\left(\text{preop weight}-\text{postop nadir weight}\right)\right]\times 100$$

Absolute recurrent weight gain (ARWG) was calculated using the following formula:

$$\left(\text{current weight}-\text{postop nadir weight}\right)$$

### 2.3 Covariates

Age at time of MBS, body mass index (BMI) at age 18 years, BMI at time of surgery, MBS procedure type, and race/ethnicity were included as covariates in all logistic regression adjusted models.

## 3 STATISTICAL ANALYSIS

Correlations between binge eating scores and RWG (absolute and percent) were determined by Pearson coefficients. RWG% was assessed in 5% increments from 5% to 50%. Each cut‐point was treated as an independent binary variable (0 = no X% RWG; 1 = yes, X% RWG) and then used as the dependent variable in separate logistic regression models.

Bivariate associations of patient characteristics with BEB and FA were determined using *t* tests, ANOVA, and chi‐square tests as appropriate. Separate multivariable logistic regression models were used to examine the adjusted associations of BEB and FA with RWG%, controlling for age at time of MBS, BMI at age 18 and time of MBS, MBS procedure, and race/ethnicity. Adjusted odds ratios (aOR) with their respective 95% confidence intervals (CI) and *p* values were reported. Odds ratios (OR) were used as measures of effect size for each logistic regression model, consistent with recommendations to report effect sizes in quantitative research to provide a comprehensive understanding of study results.^27^ The alpha was set at 0.05. SAS Studio version 9.4 was used for all analyses.

## 4 RESULTS

The mean age of the sample was 55.87 years (SD = 10.61) at the time of survey completion and 44.60 years (SD = 11.85) at the time of MBS. The mean BMI at the age of 18 years was 28.75 kg/m^2^, while the mean BMI at peak weight was 48.62 kg/m^2^. Most of the study population was female (90.48%), non‐Hispanic white (51.71%), and had at least a bachelor's degree (35.71%). The sample also included participants who identified as non‐Hispanic black (27.74%), Hispanic/Latino (14.04%), or “other race” (6.51%). The most common MBS procedure performed was a vertical sleeve gastrectomy (33.21%), followed by the Roux‐en‐Y gastric bypass (RYGB) (27.44%). The mean time since MBS was 11.1 years. The highest mean RWG was found in RYGB (26.05 kg) followed by adjustable gastric band (AGB) (24.76 kg) while the procedure with the highest RWG% was AGB (32.01%) followed by RYGB (21.89%) (Table S1). Over a third (42.3%) of participants met the criteria for BEB, 12.55% met the criteria for severe FA, and 7.36% reported behaviours and symptoms consistent with moderate and mild FA, respectively. We used 20% as the cut‐off for total body weight loss (TBWL) based on guidelines from the International Federation for the Surgery of Obesity and Metabolic Disorders.^28^

Over half (61%) of participants had <20% total body weight loss (TBWL) (Table 1). No significant difference was found between the BES and FA scores when comparing participants with ≥20% TBWL vs. <20% TBWL. Additionally, no significant associations were found between participants with BEB or FA and achieving ≥20% TBWL vs. <20% TBWL. No statistically significant difference in absolute RWG or RWG% was found between participants with bachelor's degrees vs. those with lower levels of formal education.

**TABLE 1:** Distribution of metabolic and bariatric surgery (MBS) patient characteristics (*N* = 294).

Patient characteristics	Frequency
Age (years)	
Current^a^	55.87 (10.61)
At time of surgery^a^	44.60 (11.85)
Sex, *N* (%)	
Male	28 (9.52)
Female	266 (90.48)
Race and ethnicity, *N* (%)	
Non‐Hispanic white	151 (51.71)
Non‐Hispanic black	81 (27.74)
Hispanic/Latino	41 (14.04)
Other	19 (6.51)
BMI (kg/m^2^)	
At 18 years of age^a^	28.76 (8.55)
At peak weight^a^	51.15 (11.96)
At time of surgery^a^	44.64 (17.98)
Nadir^a^	32.50 (8.48)
Current BMI^a^	33.55 (48.38)
BMI ≥30, *N* (%)	202 (68.70)
BMI ≥40, *N* (%)	83 (28.20)
MBS weight loss or gain	
Percent TBWL^a^	30.65 (14.06)
<20% TBWL%	61 (22.76)
Absolute RWG^a^	20.59 (39.87)
Percent RWG^a^	18.43 (103.11)
Time since MBS	
Years^a^	11.1 (8.2)
Recurrent weight gain %, *N* (%)	
≥5%	146 (60.83)
≥10%	129 (53.75)
≥15%	118 (49.17)
≥20%	106 (44.17)
≥25%	96 (40.00)
≥30%	89 (37.08)
≥35%	78 (32.50)
≥40%	67 (27.92)
≥45%	57 (23.75)
≥50%	48 (20.00)
MBS surgery type, *N* (%)	
Vertical sleeve gastrectomy	92 (33.21)
Roux‐en‐Y gastric bypass	76 (27.44)
Adjustable gastric band	61 (22.02)
Endoscopic sleeve gastroplasty	22 (7.94)
Biliopancreatic diversion (with duodenal switch)	4 (1.44)
Other/unsure	22 (7.95)
Binge eating behaviours, *N* (%)	
No	142 (57.72)
Yes	104 (42.28)
Food addiction, *N* (%)	
None	168 (72.73)
Mild	17 (7.36)
Moderate	17 (7.36)
Severe	29 (12.55)

Abbreviations: BMI, body mass index; MBS, metabolic and bariatric surgery; RWG, recurrent weight gain; TBWL, total body weight loss.

^a^ Mean(SD) reported.

The minimum, maximum, mean, and standard deviation (SD) response score for each question on the Yale Food Addiction Scale 2.0 (YFAS 2.0) is presented in Table 2. The highest mean score (mean = 3.70; SD = 2.31) was reported for question 11: “I tried and failed to cut down on or stop eating certain foods,” followed by question 4, “If I had emotional problems because I hadn't eaten certain foods, I would eat those foods to feel better” (mean = 3.29; SD = 2.49). The lowest mean score was observed for question 12, “I was so distracted by eating that I could have been hurt (e.g., when driving a car, crossing the street, operating machinery)” (mean = 1.30; SD = 1.03). Similar statistics for each question on the BES are presented in Table 3. The highest mean score (mean = 2.52; SD = 0.97) was reported for question 1: “Self‐conscious about body weight or size,” followed by question 4, “eating when bored” (mean = 2.46; SD = 0.90). The lowest mean score was reported for question 12, “eating in secret to avoid being shamed” (mean = 1.44; SD = 0.77). AGB had the highest BES (14.68) and FA score (2.89) followed by other for both BES (14.75) and FA (2.79) (Table S1).

**TABLE 2:** Distribution of individual Yale food addiction scale question scores.

S. no.	Question	Min, max	Mean (SD)
1	I ate to the point where I felt physically ill.	1, 8	2.44 (1.68)
2	I spent a lot of time feeling sluggish or tired from overeating.	1, 8	2.92 (1.97)
3	I avoided work, school, or social activities because I was afraid, I would overeat there.	1, 8	1.52 (1.40)
4	If I had emotional problems because I hadn't eaten certain foods, I would eat those foods to feel better.	1, 8	2.29 (1.82)
5	My eating behaviour caused me a lot of distress.	1, 8	3.29 (2.49)
6	I had significant problems in my life because of food and eating. These may have been problems with my daily routine, work, school, friends, family, or health.	1, 8	3.16 (2.54)
7	My overeating got in the way of me taking care of my family or doing household chores.	1, 8	2.18 (1.98)
8	I kept eating in the same way even though my eating caused emotional problems.	1, 8	3.00 (2.41)
9	Eating the same amount of food did not give me as much enjoyment as it used to.	1, 8	3.05 (2.40)
10	I had such strong urges to eat certain foods that I couldn't think of anything else.	1, 8	2.80 (2.07)
11	I tried and failed to cut down on or stop eating certain foods.	1, 8	3.70 (2.31)
12	I was so distracted by eating that I could have been hurt (e.g., when driving a car, crossing the street, operating machinery).	1, 8	1.30 (1.03)
13	My friends or family were worried about how much I overate.	1, 8	2.01 (1.82)

**TABLE 3:** Distribution of individual binge eating scale question scores.

S. no.	Behaviour	Min, max	Mean (SD)
1	Self‐conscious about body weight or size	1, 4	2.52 (0.97)
2	Eating quickly	1, 4	1.97 (1.04)
3	Difficulty controlling eating urges	1, 4	1.86 (0.89)
4	Eating when bored	1, 4	2.46 (0.90)
5	Eating when not hungry	1, 4	2.00 (0.72)
6	Feeling guilty after over‐eating	1, 3	1.90 (0.71)
7	Binge eating during diets	1, 4	1.72 (0.77)
8	Eating to a point of feeling stuffed	1, 4	1.83 (0.88)
9	Binge‐eating & calorie cutting cycles	1, 4	1.71 (0.93)
10	Able to stop eating when needed	1, 4	1.75 (0.85)
11	Inducing vomiting to relieve ‘stuffed feeling’	1, 4	1.68 (0.64)
12	Eating in secret to avoid being shamed	1, 4	1.44 (0.77)
13	Daily meals/eating habits	1, 4	2.15 (1.02)
14	Constant fixation on controlling food intake	1, 4	1.95 (0.98)
15	Constantly thinking about food	1, 4	1.92 (0.91)
16	Aware of the correct portion size for each meal	1, 3	1.65 (0.69)

A significant positive correlation was observed between binge eating scores and ARWG (*r* = 0.15; *p* = 0.015) but a weaker, insignificant correlation with RWG% (*r* = 0.06; *p* = 0.389). Based on the unadjusted tests of associations, we found significant associations between ARWG and age at time of surgery (*p* = 0.032), BMI at 18 years (*p* = 0.01), and current BMI (*p* = 0.001). RWG (%) was significantly associated with MBS procedure type (*p* = 0.001) and current BMI (*p* = 0.001). After adjustment, multivariable logistic regression models showed a significant association between BEB and all magnitudes of RWG (Table 4). The strongest adjusted association was seen at ≥50% RWG, where participants with BEB had 3.07 times higher odds of a ≥50% RWG compared to those without BEB [OR = 3.07; 95% CI: 1.45, 6.49; *p* = 0.003]. This was followed by the 45% RWG category [OR = 2.75; CI: 1.39, 5.45; *p* = 0.004], then 40% RWG [OR = 2.46; CI 1.31, 4.63; *p* = 0.005].

**TABLE 4:** Adjusted association between binge eating behaviours (BEB) and percent recurrent weight gain.

Recurrent weight gain (%)	Odds ratio (95% CI)	*p* value
≥5	2.52 (1.39,4.58)	0.002*
≥10	2.54 (1.44,4.49)	0.001*
≥15	2.61 (1.49,4.59)	0.001*
≥20	2.58 (1.46,4.54)	0.001*
≥25	2.50 (1.42,4.41)	0.002*
≥30	2.50 (1.41,4.46)	0.002*
≥35	2.25 (1.24,4.08)	0.008*
≥40	2.46 (1.31,4.63)	0.005*
≥45	2.75 (1.39,5.45)	0.004*
≥50	3.07 (1.45,6.49)	0.003*

*Note*: All odds ratios were adjusted for age at time of surgery, BMI at 18 years, BMI at time of surgery, type of MBS surgery, and race.

* Significant at α = 0.05.

## 5 DISCUSSION

This study reports on the prevalence of BEB and FA and their independent associations with post‐MBS RWG among a sample of patients attending an academic obesity medicine program. Results showed that there is a strong association between BEB and post‐MBS RWG. No significant association was found between FA and post‐MBS RWG. Upon secondary analysis, we found no significant difference in the association of BEB and RWG by race and ethnicity. These findings have important implications for post‐MBS patient care and identifying potential risk factors for RWG, specifically BEB.

Our findings build on existing research and confirm the association between BEB and post‐MBS RWG at various RWG% cut‐offs. Specifically, the adjusted odds ratios observed in this study for BEB and RWG% indicate moderate to large effect sizes, as per established guidelines for interpreting odds ratios.^27^ Several studies have found significant associations between pre‐MBS BED with suboptimal clinical response and increased RWG after surgery.^19^ Published data suggest that BED resolves or significantly improves soon after surgery but may return to pre‐MBS levels as early as 24 months post‐surgery.^29^, ^30^ Understanding these associations is crucial for informing clinical interventions and public health initiatives aimed at optimizing long‐term outcomes for individuals undergoing MBS.

Published data suggest similar post‐MBS results for people with FA, where those with higher average YFAS score showed suboptimal clinical response after surgery.^31^ However, contradictory findings from observational studies indicate no association between pre‐surgical FA and post‐MBS RWG.^32^ Some studies have identified a decrease in YFAS scores post‐operatively, with one particular study showing remission of FA in 93% of patients postoperatively.^32^, ^33^ This evidence could explain our findings of no association between FA and RWG, given that our subjects completed the YFAS with their postoperative symptoms and behaviours.

Additionally, the results of our study exhibited differences in post‐MBS RWG outcomes for BEB and FA, independently, providing additional evidence of the distinct nature of these eating disorders. The current literature presents FA as analogous to a substance use disorder, whereas BED or BEB is regarded as a behavioural or psychological disorder.^34^ A growing body of evidence points to physiological factors that promote RWG, including metabolic adaptation resulting from shifts in neurohormonal determinants of energy balance.^35^ The observation that BEB is linked to post‐MBS RWG% while FA does not suggest that behavioural and psychological disorders pose additional significant challenges to weight management for individuals with obesity. This highlights the importance of enhanced psychological screening and interdisciplinary collaboration while caring for post‐MBS patients who are experiencing, or may be at risk for, RWG and exhibit behaviours consistent with BEB. Individuals experiencing BEB may benefit from additional interventions such as cognitive behavioural therapy (CBT) or pharmacotherapy to improve long‐term weight outcomes.^34^ Additionally, while adjunctive lifestyle interventions have not been shown to impact recurrent weight gain when implemented immediately after bariatric surgery,^36^ individuals with BEB may benefit from such interventions at a later period, such as during the weight‐loss maintenance phase.

Strengths of this study include its large sample size, racially diverse population, and broad age ranges, which contribute to external validity through increased generalizability to the larger post‐MBS population. Furthermore, the BES has shown high sensitivity and specificity for differentiating between individuals with and without BEB across various demographics, thus adding to the precision of our study's results.^37^ It is also important to consider the specific types of behaviours that received the highest scores on the BES. While our study found a significant relationship between post‐MBS RWG and BEB, it is worth noting that the highest scores for the BES were for questions concerning body image, eating out of boredom, and meal structure. These components, while essential to understanding BED, may not exclusively represent BEB and may be present in individuals who do not have BEB. We suspect some interference by this limitation in the observed association between RWG and BEB, especially since body image concerns serve as a hindrance to successful weight loss and maintenance.^38^

There are some additional limitations to consider for this study of self‐reported patient data. The sensitive nature of weight and body‐related questions may have resulted in erroneous data reporting by participants, attributable to social desirability bias. Additionally, the use of a cross‐sectional study design prevented us from establishing causal inferences and determining temporality between each eating disorder and RWG. Furthermore, the categorization of continuous scores seen in the BES and YFAS may have decreased the precision and statistical power of the results.

Overall, this study highlights the high prevalence of symptoms and behaviours consistent with BEB following MBS, and their association with post‐MBS RWG. No association was found between FA and post‐MBS RWG, which suggests that these are distinct clinical entities that infer different health risks for MBS patients. Our data support that post‐MBS patients should be serially screened for BEB and maladaptive eating behaviours to identify those who may be at risk for suboptimal clinical response post‐MBS, RWG, adverse health outcomes; and those who may benefit from additional interventions and support.

## CONFLICT OF INTEREST STATEMENT

Dr. Jaime P. Almandoz has received advisory/consulting fees and/or other support from Novo Nordisk A/S, Boehringer Ingelheim, and Eli Lilly and Company. All other authors declare no competing financial interests.

## Supporting information


**DATA S1:** Supporting Information.

## Data Availability

The data that support the findings of this study are available from the corresponding author upon reasonable request.
