# Supplementary material for: Binge eating behaviours are associated with recurrent weight gain after metabolic and bariatric surgery
Source: Clin Obes. 2025 Jan 27;15(3):e12735. doi: 10.1111/cob.12735 (PMC12096054; doi:10.1111/cob.12735)
Supplement: Supplementary file 1 — DATA S1: Supporting Information. [file COB-15-e12735-s001.pdf]

Binge Eating Behaviors Are Associated with Recurrent Weight Gain After Metabolic and Bariatric Surgery

Matthew Cali
Medical School
University of Texas Southwestern
Dallas, TX, USA
Email: matthew.cali@utsouthwestern.edu

Deepali K. Ernest, MPH
Department of Epidemiology
University of Texas Health Science Center
School of Public Health
Houston, TX, USA
Email: deepali.k.ernest@uth.tmc.edu

Luyu Xie, PharmD, PhD
Department of Epidemiology
University of Texas Health Science Center
School of Public Health
Dallas, TX, USA
Email: luyu.xie@utsouthwestern.edu

Jeffrey N. Schellinger, MCN, RD, CSOWM
Department of Internal Medicine, Division of Endocrinology
University of Texas Southwestern Medical Center
Dallas, TX, USA
Email: jeffrey.schellinger@utsouthwestern.edu

M. Sunil Matthew, MS
Department of Epidemiology
University of Texas Health Science Center
School of Public Health
Dallas, TX, USA
Email: matthew.mathew@utsouthwestern.edu

Aparajita Chandrasekhar, MPH
Department of Epidemiology
University of Texas Health Science Center
School of Public Health
Dallas, TX, USA
Email: aparajita.chandrasekhar@uth.tmc.edu

Jane Guo, MCN, RD
Department of Clinical Nutrition
University of Texas Southwestern Medical Center
Dallas, TX, USA
Email: janeg1218@gmail.com

Gloria L. Vega, PhD
Department of Clinical Nutrition
University of Texas Southwestern Medical Center
Dallas, TX, USA
Email: gloria.vega@utsouthwestern.edu

Sarah E. Messiah, PhD, MPH, FTOS
Department of Epidemiology
University of Texas Health Science Center
School of Public Health
Dallas, TX, USA
Email: sarah.messiah@utsouthwestern.edu

Jaime P. Almandoz, MD, MBA, FTOS
Department of Internal Medicine, Division of Endocrinology
University of Texas Southwestern Medical Center
Dallas, TX, USA
Email: jaime.almandoz@utsouthwestern.edu

Table S1. Distribution of binge eating scores, food addiction scores, RWG, and RWG% by type of metabolic and bariatric surgery (MBS).

	**MBS Type**			
	RYGB	ESG	AGB	Other +
Binge Eating Score	11.67 (7.95)	10.22 (7.84)	14.68 (9.15)	14.75 (9.42)
Food Addiction Score	2.42 (2.11)	2.35 (2.40)	2.89 (2.55)	2.79 (2.39)
Mean RWG (kg)	26.05 (45.87)	16.01 (23.98)	24.76 (39.93)	15.75 (37.75)
Mean RWG%	21.89 (129.35)	19.62 (33.17)	32.01 (71.29)	8.60 (107.35)

Abbreviations: RYGB = Roux-en-Y Gastric Bypass | ESG = Endoscopic Sleeve Gastroplasty | AGB = Adjustable Gastric Band

+ Other category also includes vertical sleeve gastrectomy and biliopancreatic diversion (with Duodenal Switch)
